# Ameliorative Potentials of Cocoyam (*Colocasia esculenta* L.) and Unripe Plantain (*Musa paradisiaca* L.) on the Relative Tissue Weights of Streptozotocin-Induced Diabetic Rats

**DOI:** 10.1155/2013/160964

**Published:** 2013-07-22

**Authors:** C. O. Eleazu, M. Iroaganachi, K. C. Eleazu

**Affiliations:** ^1^Department of Biochemistry, National Root Crops Research Institute, P.O. Box 380, Umuahia, Abia State, Nigeria; ^2^Department of Food Science and Technology, Abia State Polytechnic, Aba, Nigeria; ^3^Department of Biochemistry, Michael Okpara University of Agriculture, P.O. Box 380, Umuahia, Abia State, Nigeria

## Abstract

*Aim*. To investigate the ameliorating potentials of cocoyam (*Colocasia esculenta* L.) and unripe plantain (*Musa paradisiaca* L.) incorporated feeds on the renal and liver growths of diabetic rats, induced with 55 and 65 mg/kg body weight of Streptozotocin. *Method*. The blood glucose level of the rats was measured with a glucometer, the protein and glucose and specific gravity (SPGR) in the urine samples of the rats were measured using urine assay strips and urinometer respectively. The chemical composition and antioxidant screening of the test feeds were carried out using standard techniques. *Results*. Administration of the test feeds for 21 days to the diabetic rats of groups 4 and 5, resulted in 58.75% and 38.13% decreases in hyperglycemia and amelioration of their elevated urinary protein, glucose, SPGR, and relative kidney weights. The diabetic rats administered cocoyam incorporated feeds, had 2.71% and 19.52% increases in weight and growth rates, the diabetic rats administered unripe plantain incorporated feeds had 5.12% and 29.52% decreases in weight and growth rates while the diabetic control rats had 28.69%, 29.46%, 248.9% and 250.14% decreases in weights and growth rates. The cocoyam incorporated feeds contained higher antioxidants, minerals and phytochemicals except alkaloids than unripe plantain feed. *Conclusion*. Cocoyam and unripe plantain could be useful in the management of diabetic nephropathy.

## 1. Introduction

Diabetes is one of the most challenging diseases of the 21st century that affects essential biochemical pathways of the body (carbohydrate, protein, and lipid metabolism) and whose prevalence is rising globally, including the rural Nigerian populations [1, 2]. Due to the inability of the modern therapy to control all the pathophysiological aspects of the disorder as well as the enormous cost it poses on the economy of the developing nations of the world, alternative strategies are urgently needed [3]. The use of medicinal plants in the traditional management of diabetes mellitus could play an important role in the lives of rural people, particularly in remote parts of developing countries which are poorly served with health facilities.

During diabetes, the liver has been reported to decrease in weight due to enhanced catabolic processes such as glycogenolysis, lipolysis, and proteolysis, which is the outcome of lack of insulin in the liver cells while the kidney has been reported to increase in weight due to glucose overutilization and subsequent enhancement in glycogen synthesis [4], lipogenesis, and protein synthesis. These changes could lead to serious microvascular renal complications, which involve a series of metabolic changes in the pathogenesis of diabetic nephropathy. Moreover, despite much research work, the diabetic kidney epidemic keeps increasing, and over 40% of diabetic patients worldwide have been reported to develop severe diabetic nephropathy [5]. Patients with diabetic kidney failure undergo either painful dialysis or kidney transplant [6] which is costly and harmful.

The diets/medicinal plants that are commonly used in the management of diabetes in Nigeria include acha (*Digitaria exilis*), breadfruit (*Treculia africana*), and beans (*Phaseolus vulgaris*) [7]. However, diabetic patients have often complained of the monotony of staying on a particular diet (personal communication), and this has therefore increased the research into other plants with similar antidiabetic potentials as the ones being used. 

Plantain (*M. paradisiaca*) belongs to the “Musaceae” family and it is cultivated in many tropical and subtropical countries of the world. Plantain is a source of starchy staple for millions of people in Nigeria. Unripe plantain contains low quantities of minerals and sugars. Although unripe plantain has been scientifically documented as a hypoglycemic plant [7], there is paucity of information in the literature on its use in the management of diabetic complications.

Cocoyam (*Colocasia esculenta L.*) is a herbaceous perennial plant belonging to the “Araceae” family. In most African countries, cocoyam is mainly cultivated by small-scale farmers [8]. Like many plants of the Araceae family, cocoyam grows from the fleshy corm (tuber) that can be boiled, baked, or mashed into a meal and used as staple food or snack. The corms supply easily digestible starch and are known to contain substantial amounts of protein, vitamin C, thiamine, riboflavin, and niacin and significant amounts of dietary fiber [9]. The flour of cocoyam can be used for the preparation of soups, biscuits, bread, beverages, and puddings. Cocoyam has also been reported in folklore medicine in the management of diabetes mellitus. However, there is no scientific documentation on its role in the management of diabetic complications.

Since the use of medicinal plants in the traditional management of diabetes mellitus could serve as a good alternative for the management of this disease and its complications, we decided to commence a preliminary investigation with the following objectives:

1. investigating the ameliorating potentials of unripe plantain and cocoyam on the renal and liver growths of diabetic rats induced with two different concentrations of streptozotocin (55 and 70 mg/kg body weight);
2. determining the chemical composition of cocoyam and unripe plantain flours.

## 2. Materials and Methods

### 2.1. Plant Materials

The cocoyam variety (*Colocasia esculenta L.*) known locally in Nigeria as *Edeofe* was freshly obtained at harvest from National Root Crops Research Institute, Umudike, Nigeria, while the false horn unripe plantain variety (*M. paradisiaca*) was bought from Umuahia Main Market, Abia State, Nigeria. They were authenticated in the Department of Botany, Michael Okpara University of Agriculture, Umudike, Nigeria. 

### 2.2. Chemicals

Streptozotocin (STZ), DPPH (2,2-diphenyl-1-picrylhydrazyl) radical, and standard quercetin were products of Sigma-Aldrich Chemical Company, UK. All other chemicals that were used in the experiments were bought from HosLab, Umuahia, Abia State, Nigeria, and were of analytical grade.

### 2.3. Processing of the Plant Materials

The samples were properly washed, peeled, and oven dried at 50°C for 48 hours until constant weight was obtained before being pelletized and incorporated into the rat feeds. 

### 2.4. Proximate Analysis

The moisture, crude protein, lipid, crude fibre, and ash contents of the cocoyam and unripe plantain incorporated feeds were carried out using the methods of the Association of Analytical Chemists [10]. Triplicate samples were incinerated in a muffle furnace (Thermodyn Type 1400 Furnace, Dubuque, IA, USA) at 600°C until a constant weight was obtained. The total carbohydrate content of the samples was obtained by difference (100 − (%moisture + %ash + %lipid + %crude protein)) [10]. The energy value of the test feeds was calculated from the Atwater Formula of 4, 9, and 4 by multiplying the total carbohydrate content by 4, percentage lipid by 9, and percentage protein by 4, respectively, and taking the sum of the products.

### 2.5. Phytochemical Analysis

The gravimetric method of Harbone [11] was used in the determination of the percentage alkaloid contents of the cocoyam and unripe plantain incorporated feeds while the AOAC methods (1990) were used in the determination of the flavonoid, saponin, and tannin composition of the test feeds.

### 2.6. Mineral Analysis

The atomic absorption spectrophotometer (Analyst 200, Perkin Elmer, Waltham, MA, USA) was used in the analysis of Fe, Zn, Mg, and Ca; the flame photometric method was used for the analysis of K while the molybdate method [12] was used for the analysis of phosphorous contents of the cocoyam and unripe plantain incorporated feeds.

### 2.7. Rapid Thin Layer Chromatography (TLC) Free Radical Scavenging Screening

The TLC screening of the antioxidant activity of the methanolic extracts of the cocoyam and unripe incorporated feeds was carried out using the DPPH method as proposed by Mensor et al. [13] with minor modifications. With the aid of a capillary tube, stock solutions (100 mg/mL instead of 1 mg/mL) of the extracts were spotted on a silica gel Thin Layer Chromatographic (TLC) Plate and developed with a solvent system of ethanol : methanol (90 : 10). After development, the chromatograms were dried and sprayed with a 0.3 mM solution of the stable DPPH free radical. The plates were visualized for the presence of yellow spots, and the degree of activity was determined qualitatively from observation of the yellow colour intensity. Yellow spot formed (within 30 minutes of spraying) against a purple background was taken as a positive result. Quercetin was used as the positive control for this assay.

### 2.8. Animal Experiments

#### 2.8.1. Selection of Animals

Forty male albino rats of the Wistar strain (146.76–228.74 g) obtained from the animal house of the Department of Biochemistry, University of Nigeria, Nsukka, Enugu State, Nigeria, were used for the study. The rats were kept in metabolic cages in the animal house of the Department of Biochemistry, Michael Okpara University of Agriculture, Umudike, Nigeria. The rats were acclimatized for two weeks to their diets and water prior to the commencement of the experiment and were maintained under a constant 12 h light and dark cycle and at room temperature. The experimental procedures were approved by the Ethical Committee of Michael Okpara University of Agriculture, Umudike, Nigeria. The National Institutes of Health Principles of Laboratory Animal Care [14] were observed.

### 2.9. Induction of Diabetes

Freshly prepared solution of streptozotocin (0.1 g dissolved in 5 ml of freshly prepared sodium citrate buffer 0.1 M, pH 4.5) was injected intraperitoneally to the rats at a dosage of 65 mg/kg body weight at fasting state [15]. Blood was collected from the tail vein, and the blood glucose concentration was analyzed prior to the commencement of the dietary feeding using a blood glucose meter (Double G Glucometer, USA) and subsequently, twice in a week, throughout the experiment. The STZ-treated rats with fasting blood glucose levels > 200 mg/dL after seven (7) days of induction of STZ were considered to be diabetic. The severity of diabetes was checked in the 24-hour urine samples of the STZ-treated rats using Urine Glucose Detection Strips (Clinistix, Bayer Health Care, USA) and Urine Reagent Strips for urinalysis (qualitative and quantitative) tests for glucose, protein, ketone, and bilirubin (CONDOR-TECHO URS-10, Condor-Teco Medical Technology Co., Ltd., China). The specific gravity of the urine samples was determined with a urinometer. The rats were also observed for physical activity such as excessive thirst (polydypsia) and excessive hunger (polyphagia). 

### 2.10. Experimental Procedure

The experimental rats with stable diabetic condition were then divided into 4 subgroups (groups 2 to 5) with six rats per group while the nondiabetic rats formed the first group as follows:

 group 1: normal rats administered standard rat pellets (nondiabetic control);
 group 2: diabetic control rats administered 55 mg/kg body weight STZ;
 group 3: diabetic control rats administered 70 mg/kg body weight STZ;
 group 4: diabetic rats administered cocoyam incorporated feed;
 group 5: diabetic rats administered unripe plantain incorporated feed.

Their diets and water were both administered *ad libitum* for 21 days, after which the rats were anesthetized with chloroform and their liver and kidney collected and weighed. The body weights and feed intakes of the rats were recorded on a daily basis, using an electronic weighing balance (Model Scout Pro, Ohaus Corporation, USA), and were calculated as

(1) Percentage  change  in  weight     =Initial  weight  −  Final  weight        Initial  weight    ×100,Feed  intake=Feed  administered  −  Residue,Percentage  change  in  fasting  blood  glucose  (FBG)   =  Initial  FBG  −  Final  FBG        Initial  FBG×100,Percentage  growth  rate =  Final  weight  −  Initial  weight        Experimental  duration    ×100,Relative  liver  weight  (g/100 g) =      Total  liver  weight      Final  body  weight×100,Relative  kidney  weight  (g/100 g) =  Total  kidney  weight      Final  body  weight  ×100.

### 2.11. Statistical Analysis

Data was subjected to analysis using the Statistical Package for Social Sciences (SPSS), version 15.0. Results were presented as the means ± standard deviations of triplicate experiments. One-way analysis of variance (ANOVA) was used for comparison of the means. Differences between means were considered to be significant at *P* < 0.05 using the Duncan Multiple Range Test. 

## 3. Results

The administration of STZ at dosages of 55 and 70 mg/kg body weight to the rats of groups 2 to 5 produced stable diabetic condition within 7 days in most of the experimental rats. Administration of the cocoyam incorporated feed to the diabetic rats of group 4 resulted in a 58.75% decrease in the resulting hyperglycemia while the administration of the unripe plantain incorporated feed to the diabetic rats of group 5 resulted in a 38.13% decrease in the resulting hyperglycemia compared with the diabetic controls and the non-diabetic rats (Table 1).

The diabetic rats of groups 2 to 5 had varying levels of glucose and protein in their urine by the 1st and 2nd weeks of the experimentation (Table 2) which indicated the severity of their diabetic condition. However, by the last week of the experimentation, administration of the test diets (cocoyam and unripe plantain) to the diabetic rats of groups 4 and 5 resulted in their excretion of trace/low amounts of glucose and proteins in their urine. 

The specific gravity of the urine of the diabetic rats in groups 2 to 5 was elevated, ranging from 1.06 to 1.07 by the 1st and 2nd weeks of the experimentation (Table 2). However, by the last week of the experimentation, administration of the test diets to the diabetic rats of groups 4 and 5 resulted in the amelioration of the elevated specific gravities of their urine. 

The body weights of the diabetic control rats of groups 2 and 3 as well as the diabetic rats administered unripe plantain incorporated feed decreased by 28.69, 29.46, and 5.12%, respectively. On the contrary, the body weights of the diabetic rats administered cocoyam incorporated feed increased by 2.71% compared with the non-diabetic rats administered standard rat pellets whose body weights increased by 6.21% (Table 3). 

The percentage growth rates of the diabetic control rats of groups 2 and 3 as well as the diabetic rats administered unripe plantain incorporated feed decreased by 248.9, 250.14, and 29.52%, respectively. On the contrary, the percentage growth rates of the diabetic rats administered cocoyam incorporated feed increased by 19.52% compared with the non-diabetic rats administered standard rat pellets whose percentage growth rates increased by 60.14% (Table 3).

The liver weights of the diabetic rats of groups 2 and 3 showed a significant decrease (*P* < 0.05) compared with the nondiabetic rats. In addition, there was no significant difference (*P* > 0.05) in the liver weights of the diabetic rats administered cocoyam feed and the liver weights of the two groups of diabetic control rats. However, the liver weights of the diabetic rats administered unripe plantain feed were significantly lower (*P* < 0.05) than the liver weights of the two groups of diabetic control rats (Table 3).

There were no observed significant differences (*P* > 0.05) in the kidney weights of the nondiabetic, diabetic control, and diabetic rats administered cocoyam and unripe plantain feeds, respectively (Table 3). 

The relative liver weights of the diabetic control rats administered STZ at a dosage of 55 mg/kg body weight and the diabetic rats administered unripe plantain incorporated diets were not significantly different from each other (*P* > 0.05) while the relative liver weights of the diabetic rats administered cocoyam incorporated feed differed significantly from the relative liver weights of the two groups of diabetic control rats (*P* < 0.05) (Table 3). 

The relative kidney weights of the diabetic control rats were significantly higher than those of the non-diabetic rats and diabetic rats treated with cocoyam and unripe plantain feeds (*P* < 0.05). In addition, there was no significant difference in the relative kidney weight of the diabetic rats administered cocoyam incorporated feeds and the nondiabetic rats (*P* > 0.05) (Table 3).

 The feed intake of both the experimental and nondiabetic rats increased by the last week of the experimentation (Table 5).

The feed composition that was given to group 4 diabetic rats comprised 77% cocoyam flour, 9% soya bean flour, 4% vitamin mixture, 2% salt, 4% banana flavour, and 4% groundnut oil while the feed composition that was given to group 5 diabetic rats comprised 77% unripe plantain flour, 9% soya bean flour, 4% vitamin mixture, 2% salt, 4% banana flavour, and 4% groundnut oil.

The proximate composition of the cocoyam incorporated feed indicated that it contained, on average, 3.64% moisture, 10.67% ash, 1.51% crude fibre, 3.42% lipids, 8.44% crude protein, 73.83% carbohydrate, and 359.86 Kcal/100 g of energy while that of the unripe plantain incorporated feed contained, on average, 3.41% moisture, 8.93% ash, 8.52% lipid, 9.76 protein, 69.39% carbohydrate, and 393.24 Kcal/100 g of energy (Table 6).

The Thin Layer Chromatographic screening of the methanolic/ethanolic extracts of the unripe plantain and cocoyam incorporated feeds indicated that they possessed considerable antioxidant activities, though the antioxidant activity of unripe plantain was lower than that of cocoyam as well as standard quercetin (Table 7).

**Figure 1:**
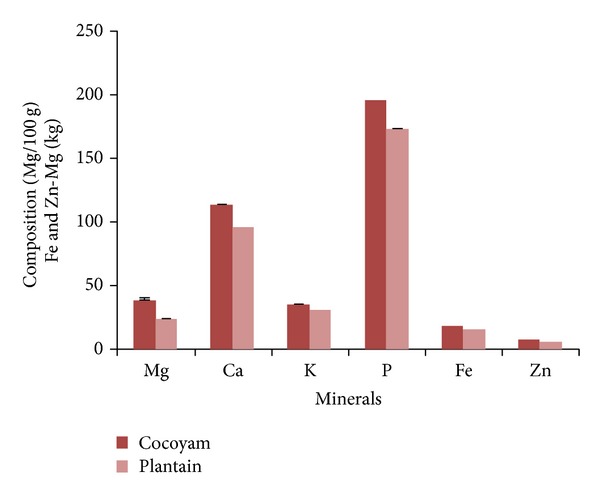
Mineral composition of cocoyam and unripe plantain incorporated feeds.

The mineral analysis of the cocoyam incorporated feeds showed that it contained, on average, 38.41 mg/100 g Mg, 113.78 mg/100 g Ca, 35.38 mg/100 g K, 195.81 mg/100 g P, 1.84 mg/100 g Fe, and 0.8 mg/100 g Zn while the plantain incorporated feed contained, on average, 23.64 mg/100 g Mg, 95.76 mg/100 g Ca, 31.48 mg/100 g K, 172.80 mg/100 g P, 1.59 mg/100 g Fe, and 0.62 mg/100 g Zn (Figure 1). 

**Figure 2:**
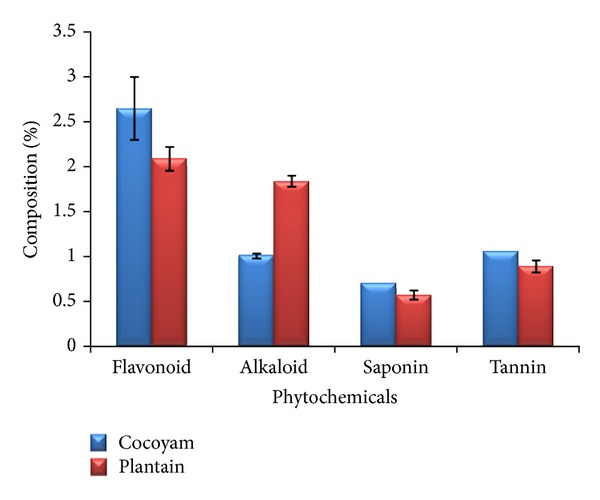
Phytochemical composition of cocoyam and unripe plantain incorporated feeds.

The phytochemical analysis of the cocoyam incorporated feed indicated that it contained, on average, 2.65% flavonoid, 1.01% alkaloid, 0.70% saponin, and 1.06% tannin while the unripe plantain incorporated feed contained, on the average, 2.09% flavonoid, 1.84% alkaloid, 0.57% saponin, and 0.89% tannin (Figure 2).

## 4. Discussion

The STZ rat model of diabetes is one of the most commonly used models of human disease [16] because it mimics many of the acute and chronic complications of human diabetes, and the model has the advantage of being highly reproducible. 

Findings from this study indicated that the incorporation of 77% cocoyam and unripe plantain into the feeds of the diabetic rats led to 58.75 and 38.13% decreases in their hyperglycemia by the last week of the experimentation, thus confirming the ability of cocoyam and unripe plantain to ameliorate hyperglycemia. 

Urinalysis is conducted in almost all disease cases because of its enormous prognostic and diagnostic significance [17]. 

The excretion of large amounts of glucose in the urine (glucosuria) of the STZ administered rats indicates that their renal threshold of glucose was exceeded since glucosuria occurs when the filtered glucose exceeds the Tm for glucose reabsorption. 

The glomerular membrane permits only very small amount of plasma proteins [18]. In 24 hr urine, 1–14 mg/dL of protein may be excreted by the normal kidney [19] while values greater than 30 mg/dL may be indicative of significant proteinuria. Diabetic nephropathy therefore occurs when proteins deposit in the glomerulus [20, 21]. Thus, the occurrence of varying levels of protein in the urine (proteinuria) samples of the diabetic rats of groups 2 to 5 by the 1st and 2nd week of the experiment suggests possibilities of glomerular complication. In addition, the low/trace amounts of detectable proteins in the urine samples of the diabetic rats administered cocoyam and unripe plantain incorporated feeds, by the last week of the experiment, suggest the ability of cocoyam and unripe plantain to ameliorate glomerular complication in diabetics, and this is a significant finding in this study.

Specific gravity (SPGR) is a urinalysis parameter that aids in the evaluation of kidney function and diagnosis of renal diseases. The kidneys of both humans and other mammals aid in the clearance of various water-soluble molecules via excretion in urine while the concentration of the excreted molecules determines the urine's specific gravity. Random urine may vary in specific gravity from 1.003 to 1.04, and 24-hour urine from normal patients may vary from 1.003 to 1.04 while 24-hour urine from normal patients may vary from 0.016 to 1.025 [22, 23]. However, the specific gravity of rats varies from 1.022 to 1.05 [24]. The elevated levels of SPGR in the urine samples of the diabetic rats of groups 2 and 3 by the 1st week of experimentation, compared with the non-diabetic rats, as observed in this study could be attributed to the elevated levels of glucose as well as protein in their urine, and this may be indicative of other substances that may have permeated the membrane of the glomerular filtrate and were dissolved in the urine. This also suggests, in addition, severe renal complications for the rats of these groups. However, the reduction in the elevated urinary SPGR values of the diabetic rats administered cocoyam and unripe plantain incorporated feeds indicates the ability of cocoyam and unripe plantain to ameliorate glomerular complication in diabetics. 

The loss of weight and the decrease in growth rates in the STZ-treated rats despite their increased feed intake, are attributed to the fact that STZ-induced diabetes is characterized by severe loss in body weight, and this reduction is due to loss or degeneration of structural proteins, as the structural proteins are known to be a major contributor to body weight. 

Although STZ is a diabetogenic agent, intraperitoneal injections of it in experimental rats have been reported to induce kidney, pancreatic, liver, and uterine tumors in laboratory animals [25]. 

Diabetic glomerular hypertrophy constitutes an early event in the progression of glomerular pathology which occurs in the absence of mesangial expansion [26]. The increase in the liver weight in proportion to the body weights of the diabetic control rats of groups 2 and 3, compared with the control, as observed in this study is attributed to increased triglyceride accumulation leading to enlarged liver as a result of increased influx of fatty acids into the liver induced by hypoinsulinemia and the low capacity of excretion of lipoprotein secretion from liver resulting from a deficiency of apolipoprotein B synthesis. The findings of this study are in agreement with those of previous researchers [15, 27]. However, the decrease in liver weights in proportion to body weights of the diabetic rats administered cocoyam and unripe plantain feeds indicates the ability of cocoyam and unripe plantain to ameliorate diabetic liver hypertrophy, and this is another significant finding in the present study. 

In addition, the increased weight of the kidney in proportion to the body weights of the STZ diabetic control rats of groups 2 and 3, as observed in this study is indicative of diabetic glomerular hypertrophy. However, the decreased weight of the kidney in proportion to the body weight of the diabetic rats administered unripe plantain incorporated feeds indicates the potentials of unripe plantain in ameliorating diabetic kidney hypertrophy while the decreased weight of the kidney in proportion to the body weights of STZ diabetic rats administered cocoyam incorporated feeds which did not differ significantly from the nondiabetic rats suggests the kidney ameliorative potentials of cocoyam in diabetics by maintaining or regenerating the renal cell histoarchitecture, and this is another significant finding in the present study. 

The results of the TLC antioxidant screening of the cocoyam and unripe plantain incorporated feeds indicate their antioxidant activities. 

The higher quantities of flavonoids, saponin, tannin, Ca, Mg, Fe, Zn, K, P, and crude fibre but lower quantities of alkaloids in the cocoyam incorporated feed compared with the unripe plantain feed are another significant finding in the present study. 

Flavonoids, alkaloids, tannins, and flavonoids, as polyphenolic compounds, have been associated with hypoglycemic activity [28]. The inhibition of the glycolytic activity of brush border enzymes by polyphenolic compounds seems to be one of the factors that stimulates hypoglycemic action in some medicinal plants [7]. In addition, flavonoids, as antioxidants, may prevent the progressive impairment of pancreatic beta cell function due to oxidative stress, thereby reducing the occurrence of diabetes. Flavonoids like myricetin, a polyhydroxylated flavonol, stimulate lipogenesis and glucose transport in the adipocytes, hence lowering blood sugar [28, 29]. The alkaloid 1-ephedrine promotes the regeneration of islets of the pancreas, following destruction of the beta cells, hence restoring the secretion of insulin and thus corrects hyperglycemia [28]. Tannins inhibit the activities of digestive enzymes such as trypsin and amylase. The tannin epigallo-catechin-3-gallate has been reported to exhibit antidiabetic activity demonstrated [29]. 

Iron influences glucose metabolism and insulin action as well as interferes with insulin inhibition of glucose production by the liver [30]. 

Magnesium is a cofactor of the glycolytic enzyme hexokinase and pyruvate kinase. It also modulates glucose transport across cell membranes [31, 32]. Zinc plays a key role in the regulation of insulin production by pancreatic tissues and glucose utilization by muscles and fat cells [33]. Zinc also influences glyceraldehyde-3-phosphate dehydrogenase in the glycolytic pathway [34]. 

Dietary fibre decreases the absorption of cholesterol from the gut in addition to delaying the digestion and conversion of starch to simple sugars, an important factor in the management of diabetes. Dietary fibre also functions in the protection against cardiovascular disease, colorectal cancer, and obesity [35]. Thus, we may not be wrong to assume that the presence of higher quantities of flavonoids, saponin, tannin, Ca, Mg, Fe, Zn, K, P, and crude fibre as well as antioxidant activity in cocoyam than unripe plantain flour could have contributed to the higher amelioration of hyperglycemia and renal growth that we observed in this study. 

## 5. Conclusion

The study showed that the use of cocoyam and unripe plantain flours in the dietary management of diabetes mellitus could be a breakthrough in the search for plants that could prevent the development of diabetic nephropathy. Finally, cocoyam flour contains higher quantities of flavonoids, saponin, tannin, Ca, Mg, Fe, Zn, K, P, and crude fibre as well as antioxidant activity but lower quantities of alkaloids than unripe plantain flour.

## Figures and Tables

**Table 1:** Fasting blood glucose of diabetic and nondiabetic rats (mg/dL).

	Week 0	Week 1	Week 2	Week 3	PC (%)
Group 1	70.67 ± 10.60	87.00 ± 7.55	92.00 ± 8.00	93.67 ± 8.50	−7.67 (increase)
Group 2	93.00 ± 1.52^b^	232.67 ± 12.36^b^	251.00 ± 12.37^b^	280.00 ± 5.29^b^	−20.34 (increase)
Group 3	107.00 ± 9.17^c^	242.00 ± 20.88^b^	229.40 ± 10.03^b^	269.50 ± 13.87^b^	−11.36 (increase)
Group 4	57.00 ± 6.54^a^	373.00 ± 126.06^b^	176.00 ± 37.56^b^	153.88 ± 30.09^b^	58.75 (decrease)
Group 5	55.00 ± 12.25^b^	210.00 ± 9.80^b^	111.00 ± 11.43^a^	129.92 ± 52.80^ab^	38.13 (decrease)

Values are given as mean ± SD. *n* = 6; ^a^ *P* < 0.05 versus diabetic control; ^b^ *P* < 0.05 in comparison with normal control within the groups (column); PC: percentage change in fasting blood glucose.

**Table 2:** Biochemical parameters in the urine of diabetic and non-diabetic rats.

	Week 0	Week 1	Week 2	Week 3
Group 1	Glucose: −ve Protein: Nil SPGR: 1.015–1.02	−ve Trace 1.02	−ve Trace 1.02–1.025	−ve Trace 1.02–1.025

Group 2	Glucose: −ve Protein: Nil SPGR: 1.02–1.025	Trace to 2+ 100 mg/dL 1.06–1.07	+ to 2+ 100–300 mg/dL 1.03–1.04	2+ 100–300 mg/dL 1.025–1.03

Group 3	Glucose: Nil Protein: Trace SRGR: 1.02–1.03	Trace to + 30–100 mg/dL 1.05–1.07	Trace to 2+ 30–100 mg/dL 1.04–1.07	2+ to 3+ 10–300 mg/dL 1.05–1.07

Group 4	Glucose: Nil Protein: Trace SRGR: 1.02–1.025	Trace to + 30–100 mg/dL 1.06–1.07	−ve to 2+ 30–100 mg/dL 1.04–1.07	−ve to trace Nil to 30 mg/dL 1.03–1.04

Group 5	Glucose: Nil Protein: Trace SRGR: 1.02–1.025	Trace to + 30–100 mg/dL 1.06–1.07	−ve to 2+ 30–100 mg/dL 1.04–1.07	−ve to trace Nil to 30 mg/dL 1.02–1.05

−ve: negative or absent; +: positive or present.

**Table 3:** Body weights of non-diabetic and diabetic rats (g).

	Week 0	Week 1	Week 2	Week 3	PG (%)
Group 1	208.37 ± 20.74	203.47 ± 19.15	205.30 ± 20.19	216.10 ± 21.86	60.14 (increase)
Group 2	189.90 ± 36.02	182.20 ± 5.57	162.91 ± 8.40	129.93 ± 5.38	−248.90 (decrease)
Group 3	192.60 ± 23.00	178.33 ± 18.60	142.63 ± 7.51	125.80 ± 4.12	−250.14 (decrease)
Group 4	164.45 ± 20.29	151.45 ± 16.33	147.40 ± 18.38	155.55 ± 14.78	19.52 (increase)
Group 5	151.00 ± 4.24	121.10 ± 2.97	113.80 ± 2.55	114.90 ± 2.69	−29.52 (decrease)

Each value in the table is the average of triplicate experiments ± std. PG: percentage growth rate.

**Table 4:** Organ weights and relative organ weights of diabetic and non-diabetic rats.

	Liver weight (g)	Kidney weight (g)	Relative liver weight (g/100 g)	Relative kidney weight (g/100 g)
Group 1 (control)	5.85 ± 0.30	1.33 ± 0.33	2.72 ± 0.16	0.61 ± 0.11
Group 2	4.29 ± 0.23^b^	1.34 ± 0.12	3.30 ± 0.02^b^	1.03 ± 0.03^b^
Group 3	4.64 ± 0.12^b^	1.31 ± 0.16	3.69 ± 0.05^a^	1.04 ± 0.09^b^
Group 4	4.55 ± 0.24^b^	1.00 ± 0.16	2.94 ± 0.17^a^	0.64 ± 0.03^c^
Group 5	3.70 ± 0.00^c^	1.00 ± 0.00	3.23 ± 0.09^b^	0.87 ± 0.02^a^

Values are presented as means ± SD. *n* = 6; ^a^ *P* < 0.05 versus diabetic control; ^b^ *P* < 0.05 in comparison with normal control; ^c^ *P* > 0.05 in comparison with normal control.

**Table 5:** Feed intake of rats (g/week).

	Week 0	Week 1	Week 2	Week 3
Group 1	112.50 ± 2.59	108.13 ± 6.91	117.73 ± 9.45	118.33 ± 7.36
Group 2	100.97 ± 1.82	116.67 ± 2.24	108.03 ± 0.87	110.60 ± 4.12
Group 3	108.30 ± 0.31	101.27 ± 2.00	109.20 ± 1.43	112.24 ± 2.25
Group 4	100.30 ± 0.99	91.45 ± 6.29	108.75 ± 0.07	121.20 ± 4.10
Group 5	78.40 ± 3.39	85.20 ± 1.70	78.50 ± 2.12	91.40 ± 3.39

Each value in the table is the average of triplicate experiments ± std. *n* = 6 rats per group.

**Table 6:** Proximate composition of cocoyam and unripe plantain incorporated feeds (%).

Parameter	MC	Ash	CF	Lipid	Crude protein	Carbohydrate	Energy value (Kcal/100 g)
Cocoyam	3.64 ± 0.11	10.67 ± 0.04	1.51 ± 0.22	3.42 ± 0.04	8.44 ± 0.03	73.83 ± 0.04	359.86 ± 0.44
Plantain	3.41 ± 0.81^a^	8.93 ± 0.00^a^	1.45 ± 0.10^a^	8.52 ± 0.00^a^	9.76 ± 0.00^a^	69.39 ± 0.00^a^	393.24 ± 0.06^a^

^a^ *P* < 0.05 versus cocoyam feed; MC: moisture content; CF: crude fibre.

**Table 7:** Free radical scavenging activities of the methanolic/ethanolic extracts of cocoyam and unripe plantain incorporated feeds using rapid DPPH TLC screening.

Plant	Antioxidant activity	Intensity of spots
Cocoyam	Moderate	+++
Unripe plantain	Moderate	++
Quercetin	Strong	+++

The degree of activity, determined qualitatively from the observation of the yellow colour intensity: moderate (++), strong (+++).
